# Mirk kinase inhibition blocks the *in vivo* growth of pancreatic cancer cells

**DOI:** 10.18632/genesandcancer.29

**Published:** 2014-09

**Authors:** Xiaobing Deng, Eileen Friedman

**Affiliations:** ^1^ Department of Pathology Upstate Medical University, Syracuse, N.Y., USA

**Keywords:** Mirk, dyrk1B, mTOR, Kras, pancreatic cancer

## Abstract

The Mirk/dyrk1B gene is upregulated and sometimes amplified in pancreatic ductal carcinomas. In poor microenvironmental conditions Mirk mediates cell survival by maintaining cancer cells in a largely quiescent, noncycling state and by decreasing toxic ROS levels through maintaining expression of a series of antioxidant genes. Premature entry into cycle, increased ROS levels, DNA damage, and apoptosis follow Mirk kinase depletion or inhibition. Mirk kinase inhibitor EHT5372 treated Panc1 spheroids lost quiescence markers coincident with an increase in cyclin A showing entry into cycle, and exhibited DNA damage, apoptosis and smaller size. EHT5372 treatment *in vivo* led to an increased fraction of Ki67 positive, cycling cells in Panc1 xenografts whose size was reduced. Pdx-1-cre LSL/KrasG12D/Ink4a/Arf null B6 mice always develop pancreatic cancer, allowing only 30% survival by 8 weeks, while each of the Mirk kinase inhibitor treated mice survived 8 weeks. Mirk inhibition led to a roughly four-fold increase in tumor αSMA-positive fibroblasts and large stromal collagen-rich infiltrates in the pancreas that can restrain tumor growth. The mTOR inhibitor RAD001 alone, or together with EHT5372, reduced pancreatic cancer size 30-fold, while the drug combination reduced the number of microscopic tumor foci 2-fold compared to RAD001 alone.

## INTRODUCTION

Pancreatic ductal adenocarcinoma is a highly lethal disease with few treatment options, and causes the death of over 40,000 people annually. About 70% of cases are initiated by mutation in the K-ras gene. Oncogenic K-ras is required for the formation of benign pancreatic tumors, their progression, and the maintenance of invasive cancers [1],[2], [3]. Mutant K-ras has been shown to activate the serine/threonine kinase Mirk/dyrk1B through the Rac1/MKK3 signaling pathway [4] so Mirk should be an active kinase during pancreatic cancer development. Metabolic stress, oncogenes such as mutant ras, and the rapid growth of tumor cells raise reactive oxygen species (ROS) levels, making ROS levels generally higher in tumor cells than in normal cells. Tumor cells with mutant ras survive the toxic stress of high ROS by becoming dependent on non-oncogenes such as antioxidant genes [5]. Mirk lowers ROS levels by increasing expression of antioxidant genes [6]. Mirk is an active kinase in pancreatic, ovarian and colon cancer cells and is an active kinase in a murine model of pancreatic cancer where Mirk restricts Hedgehog initiated Gli1 activity to the stromal compartment [7]. Significantly, Mirk maintains the viability of the most aggressive subset of pancreatic cancer cells, those that can undergo clonal growth [4], which should include the tumor stem cells. The Mirk gene is within the 660 kb core region of the 19q13 amplicon found in 12% of all primary pancreatic cancers, but in 33% of the more advanced T3 and pT4 tumors, lymph node metastases and distant metastases [8]. Mirk protein is found in 90% of resected pancreatic cancers [9]. Pharmacologic inhibition of the kinase Mirk/Dyrk1B by the small molecule inhibitor RO5454948 enabled escape from arrest in G0 quiescence, and increased ROS levels, DNA damage, and apoptosis in pancreatic cancer cells that had entered cycle [10], and escape from quiescence led to more apoptosis in cancer cells with mutant p53 in addition to low expression of G1 CDK inhibitors [11]. Similar results were seen with Mirk depletion. Mirk expression is much lower in most normal diploid cells [12] than in cancer cells. In addition, direct measurement of Mirk kinase activity showed that kinase activity was much more elevated in ovarian cancer cells than in normal diploid ovarian epithelial cells [11]. The viability of normal diploid fibroblasts and epithelial cells was not affected by Mirk depletion [13] or by Mirk kinase pharmacologic inhibition [11]. The current study tested whether Mirk kinase could be targeted *in vivo* in xenografts of Panc1 cells with an amplified Mirk gene and in a genetic model of pancreatic cancer with no known Mirk amplification.

## RESULTS AND DISCUSSION

### Mirk kinase depletion or inhibition leads to DNA damage, increased ROS levels, and apoptosis

We speculated that inhibition of Mirk kinase might be effective in murine models of pancreatic cancer in which the Mirk gene was amplified, as it is in 12% of all primary pancreatic cancers, and in 33% of metastases [8]. Panc1 cells express the Mirk gene amplified several fold and can grow as xenografts in athymic mice, so were chosen as a model. In response to poor micro-environmental conditions, Mirk kinase phosphorylates the cell cycle regulators cyclin D1, cyclin D3, p27, and LIN52 to make cells quiescent, so inhibiting Mirk allows cells to exit quiescence even though they remain in poor conditions, like serum-starvation [10], [11]. Mirk destabilizes cyclin D isoforms [14], [13], stabilizes the CDK inhibitor p27 which is elevated in quiescent cells [6], [9], [15], and phosphorylates LIN52-ser28, required for the assembly of the quiescence-maintaining DREAM complex that includes p130/Rb2 [16]. In earlier studies depletion of Mirk allowed SU86.86 pancreatic cancer cells to escape quiescence by disassembly of the DREAM complex, causing loss of sequestered E2F4 and entry into S phase even though cells remained serum-starved [6].

**Fig.1:**
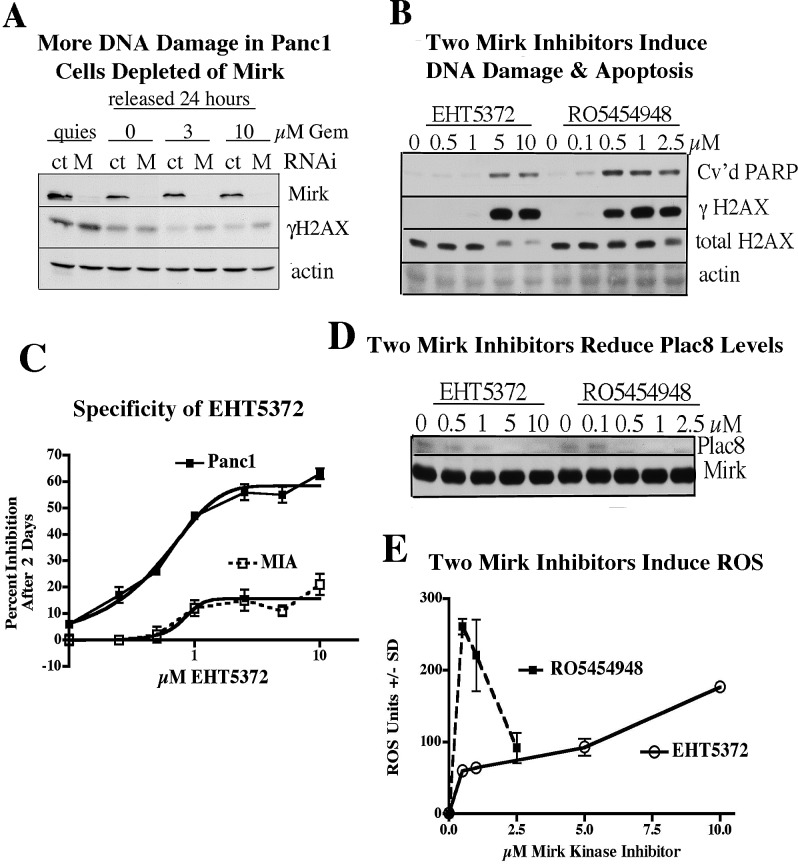
Mirk kinase depletion or inactivation by either of two inhibitors in Panc1 cells leads to DNA damage, apoptosis and increase in reactive oxygen species A. Panc1 cells were made quiescent by serum-starvation in DMEM containing 0.2% FBS for 3 days after transient transfection of synthetic RNAi duplexes to Mirk. The RNAi 5′- GTGGTGAAAGCCTATGATCAT-3′ (Invitrogen) targeted a sequence in Mirk exon 5. Cells were released from quiescence by replating after 5-fold dilution into fresh medium containing 0, 3 or 10μM gemcitabine and cultured for 24 hours. Lysates were examined by western blotting for histone H2AX phosphorylation as a measure of DNA breaks, Mirk and actin. B. Panc1 cells were made quiescent by serum-starvation for 2 days in DMEM containing 0.2% FBS while the cells were treated with increasing concentrations of the Mirk kinase inhibitors EHT5372 and RO5454948. Lysates were examined by western blotting for H2AX phosphorylation, total H2AX, the apoptotic marker cleaved PARP, and actin. C. Specificity of EHT5372: 105 cells/well per 6 well dish of Panc1 cells with abundant expression of Mirk protein or MIA cells without Mirk expression were plated, treated with the Mirk kinase inhibitor EHT5372 for 2 days in growth medium (DMEM+7% FBS), then relative cell number measured by MTT metabolism, mean +/−SD shown, n=3, with curves drawn by Boltzmann sigmoidal curve fitting. D. Panc1 cells were made quiescent by serum-starvation for 2 days in DMEM containing 0.2% FBS while the cells were treated with increasing concentrations of the Mirk kinase inhibitors EHT5372 and RO5454948. Lysates were examined by western blotting for Plac8 and for Mirk. E. Four parallel sets of Panc1 cells were grown under adherent conditions for 3 days with a range of concentrations of the Mirk kinase inhibitors EHT5372 and RO5454948. Two sets of these cells were then incubated with 5μM CM-H2DCFDA. ROS metabolism of this fluorochrome was measured, and the values minus dye were subtracted, n=4.

Mirk was effectively depleted from Panc1 cells made quiescent by serum-starvation (Fig.1A). DNA damage was shown in the serum-starved cells by increased levels of H2AX phosphorylated at S349 (γH2AX), with slightly more in the Mirk-depleted cells. Histone protein H2AX molecules become phosphorylated on serine-139 near their carboxyl terminus when they are within the chromatin at a double-stranded DNA break site, and create a focal site for DNA repair within a short time of DNA damage [17]. The Mirk-depleted cells were then released from quiescence by replating at lower density in growth medium. Half of the cultures were treated with toxic levels of the chemotherapeutic drug gemcitabine to induce DNA damage. Although gemcitabine killed many cells, the remaining cells exhibited more DNA damage if they were Mirk-depleted as well (Fig.1A). Thus Mirk depletion in Panc1 cells stressed by poor culture conditions or a chemotherapy drug correlates with more DNA damage. Similarly, Panc1 cells treated with a range of concentrations of either of two Mirk kinase inhibitors, RO5454948 or EHT5372, showed increased DNA damage detected by antibody to phosphorylated H2AX as well as increased amounts of the apoptosis marker cleaved PARP (Fig.1B). RO5454948 is not stable in rodents, so the Mirk kinase inhibitor EHT5372, with greater stability (Methods) was tested. ROS species are known to increase DNA damage, and Mirk lowers ROS levels by increasing expression of a series of at least 9 antioxidant genes including superoxide dismutase 2 and ferroxidase [6], probably through its transcriptional co-activator activities [18], [19]. Both EHT5372 and RO5454948 increased ROS levels in a dose-dependent manner (Fig.1E), as the Mirk inhibitor RO5454948 did in an earlier study [10]. The concentrations that induced the highest ROS levels also induced more DNA damage and more apoptosis, with 5 and 10μM EHT5372 and 0.5 and 1μM RO5454948 being optimal.

EHT5372 showed selective inhibitory effect on Mirk protein in a screen of over 300 protein kinases [20]. However, how much of the growth inhibitory effect on Panc1 cells was due to targeting Mirk, and not off-target kinases? We compared the growth of Panc1 cells with MIA cells, which have been reported not to express Mirk [9]. The EHT5372 EC50 was 1.2μM on Panc1 cells, but could not be determined on MIA cells, as levels from 1 to 10μM inhibited MIA growth about 10%. (Fig.1C). Thus EHT5372 targeted other kinases than Mirk within MIA cells, but this off-target effect only led to a minor effect on growth. A second test of the specificity of the EHT5372 was to determine whether the inhibitor reduced expression of Plac8, placenta specific antigen. This protein has been shown to suppress pancreatic cancer formation by blocking autophagy [21]. In prior studies Mirk depletion had been shown to reduce Plac8 expression about 5-fold [19]. Panc1 cells exhibit low levels of Plac8 [21]. However, treatment of these cells with either EHT5372 or RO5454948 markedly decreased Plac8 expression (Fig.1D). These two studies indicate that EHT5372 phenocopies Mirk depletion, and that growth inhibition by this inhibitor is primarily due to inhibition of Mirk kinase.

### Inhibition of Mirk kinase by EHT5372 has a role in inducing apoptosis of spheroid cells

**Fig.2:**
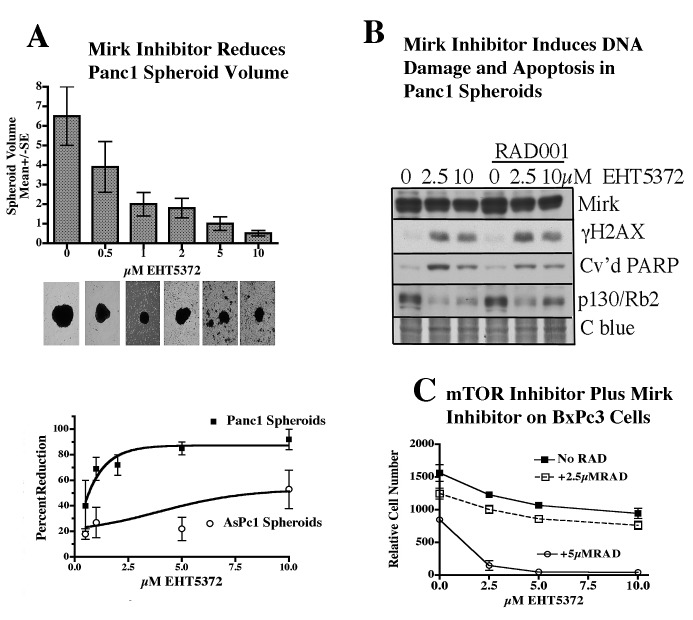
Panc1 spheroids respond to a Mirk kinase inhibitor by DNA damage, apoptosis, loss of the p130/Rb2 quiescence marker and a decrease in spheroid size A. Panc1 cells were allowed to form spheroids after plating onto nonadherent plates. The spheroids were treated with a 0-10μM Mirk kinase inhibitor EHT5372 for 11 days. Random microscopic fields were analyzed for spheroid size by measurement of the long and short axis of spheroids (n=10) and calculation of volumes = width squared x length/2. Mean+/−SE plotted. Photomicrographs of representative Panc1 spheroids are shown below the respective data columns. (below) The data for Panc1 spheroids and AsPc1 spheroids treated identically were plotted +/−SE, and curves drawn by Boltzmann sigmoidal curve fitting. B. Panc1 spheroids were treated for 7 days with the Mirk kinase inhibitor EHT5372 at 2.5 or 10μM, or the mTOR inhibitor RAD001 at 10μM, alone or with the Mirk inhibitor, before analysis by western blotting for H2AX phosphorylation as a measure of DNA breaks, the apoptotic marker cleaved PARP, and the quiescence marker p130/Rb2. The loading control is a band stained with coomassie blue. C. BxPC3 cells were seeded at low density in 24 multiwall plates, allowed to grow for 2 days, then treated for 1 day with 0-10μM EHT5372, alone or with 2.5μM or 5μM RAD001, or RAD001 alone. Mean +/−SD (n=3), one of duplicate experiments with similar data shown.

Tumor cells grown attached to typical tissue culture plates have a limited ability to model tumor cells *in vivo*. Tumor cells in spheroids, non-adherent multicellular aggregates, are highly resistant to chemotherapy, compared with tumor cells grown attached, and may better reflect drug sensitivity *in vivo* [22], [23], [24], and thus enable selection of a dosage that might be effective in animal models. Panc1 cells formed into spheroids when cultured in ultra-low attachment plates (Figs. 2A&B). Mirk protein was expressed in Panc1 spheroid cells (Fig.2B). Treatment with 10μM of the Mirk kinase inhibitor EHT5372 led to a 13-fold decrease in Panc1 spheroid size, as measured from photomicrographs, and increased the number of single cells (Fig.2A), with a dose-dependent decrease in spheroid size seen at 1μM and above. AsPc1 spheroids, with no Mirk gene amplification, required much higher levels of the inhibitor to reduce spheroid volume (Fig.2A). Panc1 spheroids were treated for 7 days with the Mirk kinase inhibitor EHT5372 alone, or together with the mTOR inhibitor RAD001. In Panc1 spheroid cells Mirk inhibition led to 10-fold more DNA damage as shown by increased levels of γH2AX, and about 6-fold more apoptosis as assayed by cleavage of PARP (Fig.2B). In earlier studies RAD001 increased the toxicity of EHT5372 towards ovarian ascites cancer cells from a series of patients, as well as ovarian cancer cell lines [25]. However, addition of RAD001 had little effect on Panc1 spheroids (Fig.2B). Thus the Mirk kinase inhibitor reduced the number of Panc1 cells in the spheroids, consistent with the cells in the outside layers dying of apoptosis and reducing spheroid volume. Interestingly, others have reported that stable short hairpin RNAs against Mirk/dyrk1B prevented the A498 renal carcinoma cell line from forming spheroid cultures [26]. Disruption of spheroids to single cells is a step in their dissolution.

In contrast to the results with Panc1 cells, in BxPc3 pancreatic cancer cells with no Mirk gene amplification, the combination of 5μM RAD001 and 10μM EHT5372 killed almost all cells (Fig.2C), while cell growth was inhibited only about 50% by 5μM RAD001 alone (data not shown). Possibly RAD001 increased the amount of DNA damaging ROS released by Mirk kinase inhibition [27], or increased expression of Mirk in BxPc3 cells [27], or both.

### Mirk kinase inhibition allows Panc1 spheroid cells cultured *in vitro* as spheroids or Panc1 cells grown as xenografts *in vivo* to inappropriately enter cycle

**Fig.3:**
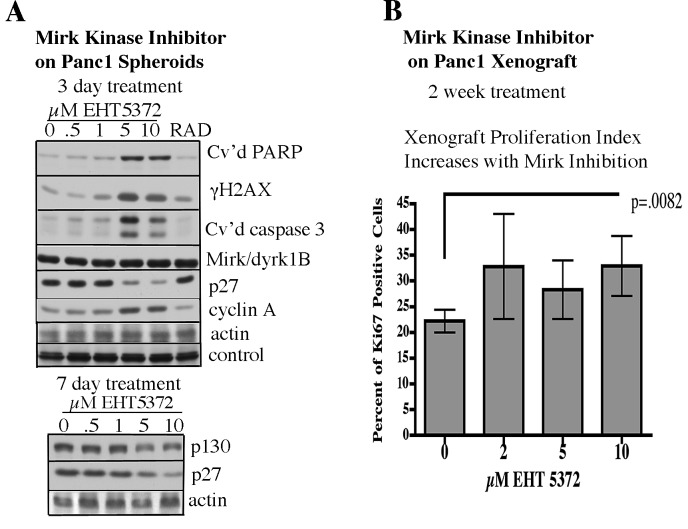
Mirk kinase inhibitor treatment *in vitro* or *in vivo* leads to increased entry into cell cycling, and DNA damage and apoptosis A. Panc1 spheroids were treated 3 or 7 days with the Mirk kinase inhibitor 0.5-10μM EHT5372 or 10μM RAD001 before analysis by western blotting of quiescence proteins p130/Rb2 and p27, cyclin A as a measure of entry into cycle, histone H2AX phosphorylation as a measure of DNA breaks, the apoptotic marker cleaved PARP and cleaved caspase 3, and for blotting controls actin and a cross-reacting band to confirm equal loading. B. 4 week old J:NU athymic mice (Jackson Labs) were injected subcutaneously under the backskin with 1 million viable Panc1 cells, 5 mice per group. After 3 weeks, palpable tumors were detected, and mice were subjected to twice weekly 0.1ml intraperitoneal injection with Mirk/dyrk1B inhibitor EHT5372 to give a final concentration of 2, 5 or 10μM (4mg/kg), or diluent over a two week period. Tumors from each group of mice were fixed, and stained for the proliferation marker Ki67, a DNA polymerase subunit. Sections containing all of the tumors in each group were photographed with the same contrast, and printed. Each print was “gridded”, and all of the cells within 4 alternate grid boxes were counted to eliminate counter bias. 454+/−21 cells were counted per treatment. Comparison of Ki67 percents by unpaired two-tailed t-tests for control vs. 10μM EHT5372 was statistically significant, p=0.0082.

Earlier studies showed that spheroids formed from ovarian cancer cells are largely in a quiescent, dormant state, expressing elevated levels of the quiescence markers p130/Rb2 and the CDKI p27 compared to cycling adherent cells, and about 85% of the spheroid cells were in G0/G1 by flow cytometry [28]. Likewise, Panc1 spheroids exhibited the quiescence marker p130/Rb2 and the CDK inhibitor p27, which serves to block cell cycling (Fig.3A). Blocking the Mirk contribution to cell quiescence allows some cancer cells to enter cycle although remaining in poor culture conditions [10], [29]. In prior studies, Mirk kinase inhibition by RO5454948 forced more SW620 colon cancer cells to enter cycle as assayed by an increase in BrdU-incorporating cells [10], and Mirk kinase depletion enabled serum-starved HD6 colon cancer cells to leave quiescence and traverse G1 to the G0/G1 boundary with S phase [13]. Likewise, inhibition of Mirk kinase reduced the quiescence marker p130/Rb2 about 5-fold in Panc1 spheroid cells (Fig.2B, Fig.3A). The DREAM complex component p130/Rb2 sequesters transcription factors necessary for cell cycling [30], [31]. Significantly, EHT5372 at 5 and 10μM strongly reduced p27 levels, while at the same time increased levels of cyclin A, a marker of cells in S phase (Fig.3A), showing movement into cycle. Thus EHT5372 inhibition of Mirk kinase enabled more Panc1 cells grown in three-dimensional culture as spheroids to leave the quiescent state and enter cycle although they remained in serum-limited culture conditions. The loss of quiescence proteins and increase in cyclin A by EHT5372 at 5 and 10μM paralleled an increase in DNA damage as shown by increased levels of γH2AX, and the apoptosis markers cleaved caspase3 and cleaved PARP (Fig.3A), showing that inappropriate entry into cycle correlated with DNA damage, apoptosis and the resulting cell loss in these Panc1 spheroids.

Likewise, inhibition of Mirk/dyrk1B *in vivo* led to inappropriate entry into cycle and cancer cell loss. Athymic mice bearing palpable tumors 3 weeks after injection of 1 million Panc1 cells were subjected to twice weekly 0.1ml intraperitoneal injection with Mirk/dyrk1B inhibitor EHT5372 to give a final concentration of 0.8mg/kg, 2mg/kg, or 4mg/kg, equivalent to 2, 5 or 10μM, or with diluent over a 2-week period. Tumors from each group were dissected free of other tissues, weighed and then fixed and stained for the proliferation antigen Ki67. Many pancreatic cancer cells *in vivo* are quiescent in G0 [32]. G0 cells do not express the proliferation related antigen Ki67. Only about 22% of Panc1 xenograft cancer cells were Ki67 positive and thus in cycle (Fig.3B, 0 lane). EHT5372 treatment increased the fraction of Ki67 Panc1 tumor cells at every concentration tested, 2, 5 or 10μM (Fig.3B). However, comparison of the fractions of Ki67 positive cells by unpaired two-tailed t-test showed only the 10μM EHT5372 value was statistically greater than control, p=0.0082 (Fig.3B). Thus Mirk kinase inhibition increased quiescent pancreatic cancer cell entry into cycle *in vivo*.

### The Mirk kinase inhibitor EHT5372 reduces the growth of Panc1 pancreatic cancer xenografts in a dose-dependent manner

**Fig.4:**
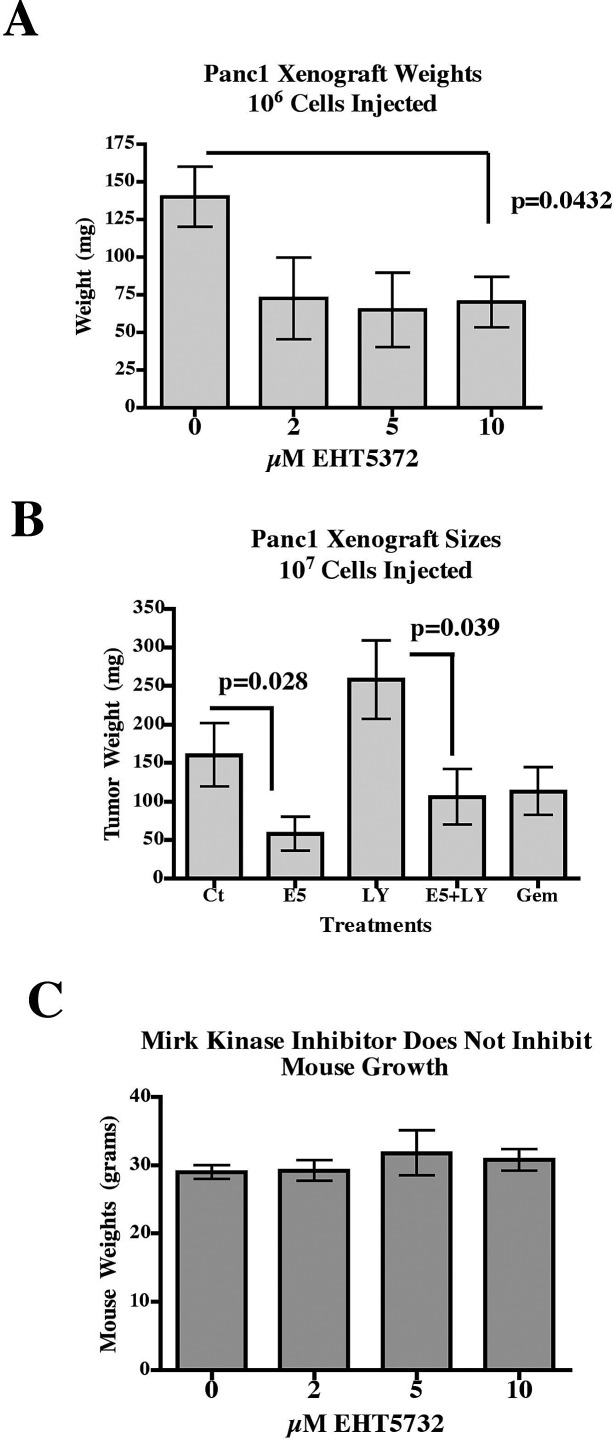
Mirk kinase inhibitor reduces the size of Panc1 xenografts in athymic mice A. Xenograft experiment described in Fig.3B. The weight of the tumors in mg shown, after dissection free of extraneous tissue. Comparison of mean tumor weights by unpaired two-tailed t-tests for control vs. 10μM EHT5372 was statistically significant, p=0.0432 B. 7 week old J:NU athymic mice (Jackson Labs) were injected subcutaneously under the backskin with 10 million viable Panc1 cells, 5 mice per group. After 10 days, palpable tumors were detected, and mice were subjected for 2 weeks to twice weekly 0.1ml intraperitoneal injection to give respectively, 4 mg/kg Mirk/dyrk1B inhibitor EHT5372, 25mg/kg gemcitabine, 4.4 mg/kg of the Chk1 inhibitor LY2603618, diluent or combinations as indicated. Tumor weight is given as mean+/−SE. Comparison of tumor weights by unpaired two-tailed t-tests for control vs. EHT5372 was statistically significant, p=0.0277, and for LY vs LY+EHT5372 was 0.0388, also statistically significant. C. After treatment, the weights of the mice in panel A were measured, mean+/−SE.

In the xenograft experiment shown in Fig.3B, tumors from each group of mice were weighed, and only 10μM EHT5372 statistically reduced tumor size (Fig.4A). This experiment was repeated with a 10-fold increase in the number of Panc1 cells injected and addition of two other drugs (Fig.4B). Pharmacological inhibition of Mirk kinase allows quiescent tumor cells with low or absent expression of CDKN2A (p16) to escape quiescence even under suboptimal culture conditions and enter cycle prematurely with unrepaired DNA damage, where some undergo apoptosis [11]. We speculated that an increase in apoptosis might occur if additional checkpoints were inactivated. In an initial survey with the Chk1 inhibitor LY2603618, a combination of this agent and the Mirk kinase inhibitor EHT5372 led to complete dispersal of the Panc1 spheroids (data not shown). Athymic mice bearing palpable Panc1 pancreatic cancer xenografts were subjected for 2 weeks to twice weekly 0.1ml intraperitoneal injections to give respectively, 4 mg/kg Mirk/dyrk1B inhibitor EHT5372, 25 mg/kg gemcitabine, 4.4 mg/kg Chk1 inhibitor LY2603618, PBS diluent or combinations as indicated. Gemcitabine had no effect. However, the short duration of treatment with the Mirk kinase inhibitor was sufficient to reduce tumor growth 3 fold (Fig.4B). Comparison of tumor weights by unpaired two-tailed t-tests for control vs EHT5372 showed that the differences were statistically significant, p=0.0277. Unexpectedly, the Chk1 inhibitor LY2603618 increased tumor growth about 60% over control values, as the tumors appeared more vascularized, but addition of the Mirk kinase inhibitor reduced tumor size about two-fold with a statistical difference, p=0.0388 for LY2603618 vs. LY2603618 and EHT5372. The Chk1 inhibitor was expected to decrease tumor growth, not increase it, so the finding that the Mirk inhibitor reduced tumor growth even in the presence of LY2603618 strengthens the observation that Mirk kinase inhibition by itself is enough to decrease the *in vivo* growth of pancreatic cancer cells known to contain a highly amplified Mirk gene and elevated levels of active Mirk kinase [9]. Thus the Mirk kinase inhibitor blocked the xenograft growth of pancreatic cancer cells with an amplified Mirk gene under two different experimental conditions.

Mirk expression levels are very low in most normal cell types except for skeletal muscle [12] where it blocks ROS generated by muscle contraction [33], suggesting that this kinase has a non- critical function in most normal cells. Supporting this interpretation, the weights of mice treated with EHT5372 were unchanged, with similar weights seen in mice treated with 2, 5, or 10μM drug or diluent only (Fig.4C). The treated mice were lively, and showed no apparent distress, or differences in behavior compared to control mice. No other assays for viability were performed.

### Mirk kinase inhibition maintained mouse viability in a genetic model of pancreatic cancer

**Fig.5:**
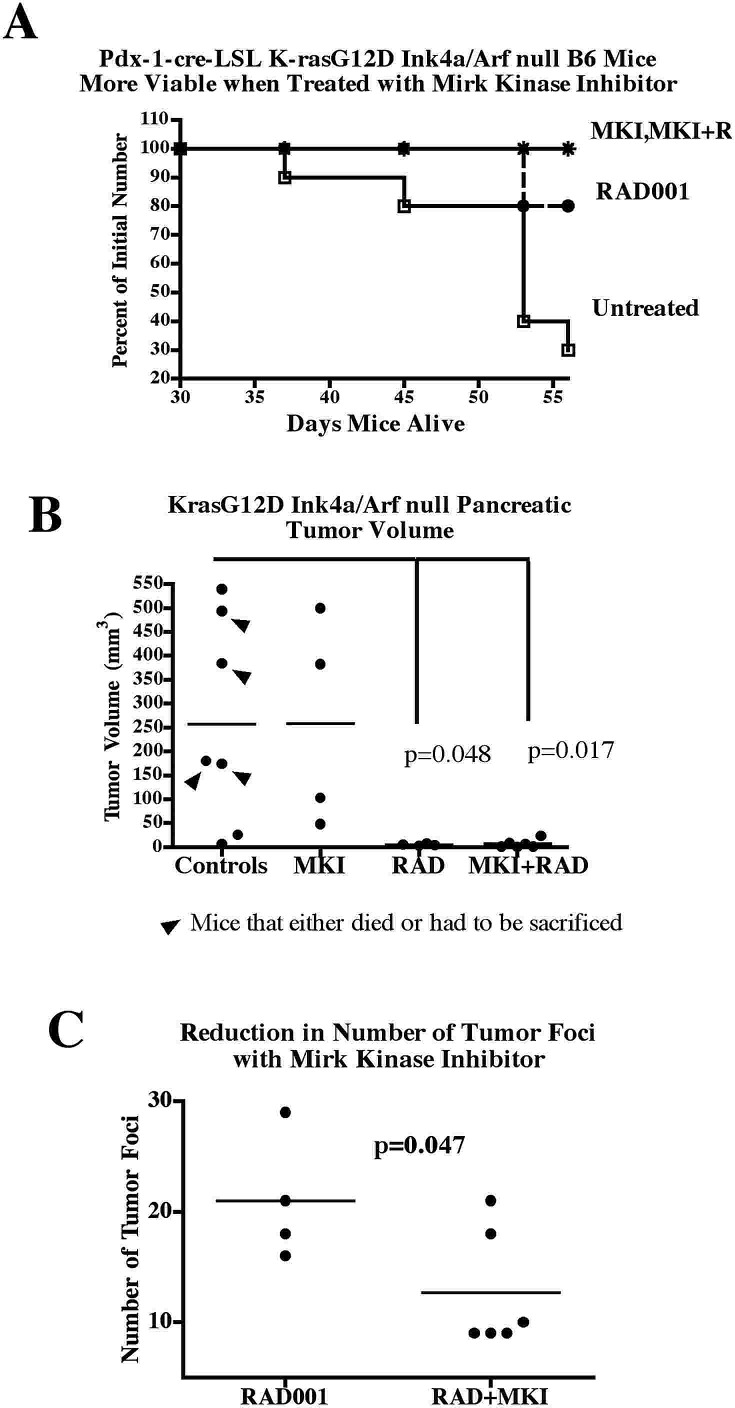
Treatment with Mirk inhibitor EHT5372 maintains the viability of Pdx-1-cre LSL/KrasG12D/Ink4a/Arf null B6 mice for 8 weeks, but leads to extensive stromal growth in the p16 null background and thus large tumors, while the addition of the mTOR inhibitor RAD001 or RAD001 alone reduces size of pancreatic cancers A. Pdx-1-cre LSL/KrasG12D/Ink4a/Arf null B6 mice were genotyped at weaning, then injected twice weekly with 4 mg/kg Mirk/dyrk1B inhibitor (MKI) EHT5372 (4 mice), EHT5372 plus 5 mg/kg RAD001 (6 mice), 5 mg/kg RAD001 (5 mice), or left untreated (10 mice) until sacrifice at 8 weeks. The days mice survived are plotted. B. The volume of the pancreatic cancers arising in each mouse was determined from width2 x length/2 of all tumors detected in an H&E section, and the total volume per mouse was added together. Only the tumors in untreated mice that lived to 52- 56 days are included. Mean+/−SE shown. The control untreated values are statistically different from the EHT5372+RAD001 values, p=0.0166, and different from the RAD001 only values, p=0.0483, both 2 tailed unpaired t tests. The mean +/−SE sizes of the tumors in cubic mm were 257+/−82, 258+/−109, 4.8+/−1, 6.6+/−4 for untreated, MKI, RAD, MKI+RAD, respectively. C. The total numbers of tumor foci in the H&E sections from the Mirk kinase inhibitor (MKI) plus RAD001 and the only RAD001 treated Pdx-1-cre LSL/KrasG12D/Ink4a/Arf null B6 mice after 8 weeks were counted. The number of foci includes the tumors of 1mm of greater, as well as the foci, which could only be seen using a microscope.

A genetically defined murine model of pancreatic cancer pairs deletion of the CDK inhibitor p16 gene with cre-lox activation of a mutant Kras gene localized to the pancreas by the Pdx-1 transcription factor [34], and such tumors express Mirk protein (Supplementary Fig.1). Pdx-1-cre LSL/KrasG12D/Ink4a/Arf null mice were bred, genotyped at weaning, then either treated with EHT5372 alone or left untreated for eight weeks when all mice should show tumor growth [34], or until death or sacrifice because of ill health. More mice were added to the untreated group, 10 in all, because only three control mice lived eight weeks, while four others either died on day 52 or 53, or had to be sacrificed at 53 days because of ill health (Fig.5A). In Fig. 5B tumors from mice that died before 8 weeks or were sacrificed due to poor health are indicated with arrowheads. The remaining three control mice died at 45 days or earlier. These control mice were not undersized compared to their littermates, as Pdx-1-cre LSL/KrasG12D/Ink4a/Arf null mice that were undersized and ill-appearing from an early age were not included in any of the groups. In marked contrast, all 10 of the mice treated with the Mirk kinase inhibitor (MKI) EHT5372 lived for the entire 8 weeks (Fig.5A).

**Fig.6:**
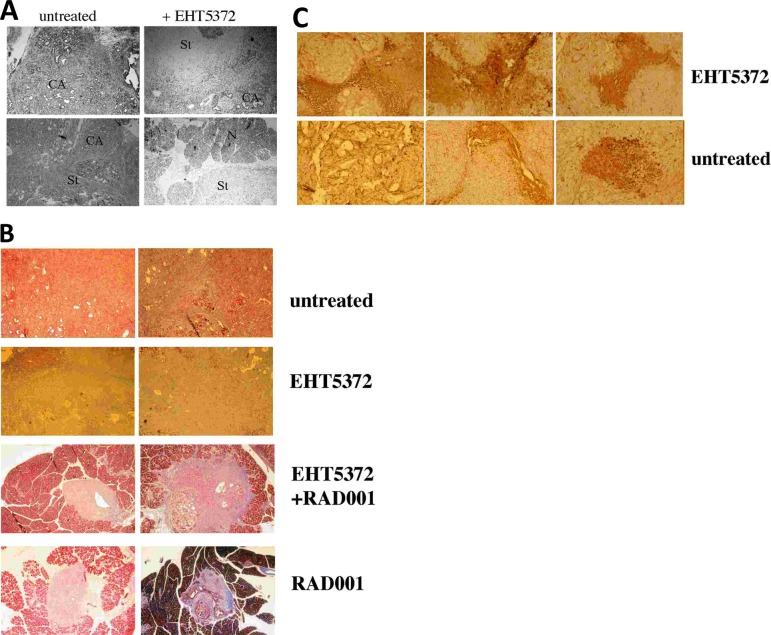
Pancreatic tumors in mice treated with Mirk/dyrk1B inhibitor EHT5372 have more collagen- containing stroma than tumors from mice left untreated until sacrifice at 8 weeks 40x magnification. A. Hematoxylin & eosin staining, with panels of untreated mice at left, and treated with EHT5372 at right. CA, focus of cancer cells, St, stroma, N, normal pancreatic acini. B. The four tumor groups were sectioned and stained with Masson's trichrome stain at the same time, and processed identically, with collagen showing a blue-green. The foci in the EHT5372+RAD001 and RAD001 only treated mice are surrounded by normal pancreatic acini. C. SMA (alpha smooth muscle actin) staining, a fibroblast marker, of sections of EHT5372-treated tumors was more intense than in untreated tumors.

When the pancreases were excised, those from the untreated group were enlarged and felt hard, compared to the pancreas from a genetically more normal littermate (Pdx-1-cre/p16null). The sectioned pancreases were stained with hematoxylin & eosin to reveal structure. The width and length of all tumors in each pancreas was measured from H&E slides using a 10x magnifying loop, and the total volume calculated. Surprisingly from the viability data (Fig.5A), a similar mean tumor size was seen in the pancreases excised from mice treated with the Mirk kinase inhibitor and the untreated controls (Fig.5B). The two largest tumors from the untreated mice and from the EHT5372-treated mice were compared by microscopy (Fig.6). The pancreases from untreated mice were often almost completely taken over by tumor with some stromal component (Fig.6A&B). However, each pancreas from the Mirk inhibitor treated mice had some tumor with a large solid core of stroma (Fig.6B) surrounded by normal-appearing tissue (Fig.6A), possibly enough normal pancreas to enable these mice to live 8 weeks. The stoma was collagen-rich and more extensive than that found in the tumors from the untreated mice (Fig.6B). Sonic hedgehog signaling induces stromal expansion in the pancreas, but is blocked in the pancreatic epithelial cells by Mirk/dyrk1B [7], [35]. Possibly inhibiting Mirk kinase activity in the pancreatic cancer cells enabled more sonic hedgehog signaling and thus more stromal growth. Also, inhibition of Mirk might push the p16 null fibroblasts into cycle. About 10% of normal diploid fibroblasts, when accumulated in G0 quiescence by serum-starvation, responded to Mirk kinase inhibition by entering cycle and moving to G2 [11]. In this murine model, the stromal fibroblasts were p16 null, so might respond more strongly to Mirk kinase inhibition, so there might be a larger population to respond to signals from the pancreatic cancer cells to make stroma. To test this hypothesis, the fibroblast content in tumors was measured by immunohistochemistry for the fibroblast marker alpha smooth muscle actin (SMA). The Mirk inhibitor treated tumors had many more fibroblasts than the untreated tumors (Fig.6C). Comparison of an equal number of sections by densitometry showed that the EHT5372-treated tumors had roughly four times the number of SMA+ fibroblasts as the controls, consistent with the increased stroma. Desmoplasia in pancreatic cancer was thought to increase aggressiveness. However, recent studies employing myofibroblast depletion have shown a strong protective role by stroma in this cancer [36], [37], which might explain the enhanced viability of the Mirk kinase inhibitor treated mice.

### Mirk kinase inhibition plus mTOR inhibition strongly reduced pancreatic cancer size in the genetic model

In our earlier studies, the combination mTOR inhibitor RAD001 and EHT5372 killed over 90% of ovarian cancer ascites taken directly from patients and maintained as spheroids in culture [25]. In the current study RAD001 did not increase the toxicity of the Mirk kinase inhibitor EHT5372 towards Panc1 spheroid cells (Fig.2B), but RAD001 did increase its toxicity towards BxPC3 cells, which have no Mirk gene amplification (Fig.2C). Possibly RAD001 would enhance the toxicity of EHT5372 in the genetic mouse model with no known Mirk amplification.

Pdx-1-cre LSL/KrasG12D/Ink4a/Arf null mice were bred, genotyped at weaning, then either treated with RAD001 alone or with RAD001 and EHT5372 for eight weeks, or until death. All of the mice treated with EHT5372 and RAD001 were viable for 8 weeks, as were all but one of RAD001- treated mice (Fig.5A). All of the mice treated with RAD001 or with both the Mirk kinase inhibitor and the mTOR inhibitor RAD001 had detectable pancreatic cancer at 8 weeks, but only as small nodules. When the total tumor volumes were calculated both groups had tumors that were over 30 times smaller than tumor volumes in the control, untreated mice (Fig.5B). Interestingly, there was no enhanced stromal growth when the mTOR inhibitor was added with the Mirk kinase inhibitor (Fig.6B). Examination of the slides from the RAD001-only treated mice showed that these pancreases had a large number of microscopic tumor foci that were too small to be measured for tumor volume. These were about twice as many tumor foci in the pancreases from mice treated with only RAD001 compared with the pancreases from mice treated with RAD001 and the Mirk kinase inhibitor (Fig.5C), suggesting that at a time later than 8 weeks, the RAD001-treated mice would have more pancreatic tumors than the mice treated with both inhibitors. Thus the Mirk kinase inhibitor reduced the number of minute tumors when added together with the mTOR inhibitor, and this drug combination strongly reduced pancreatic cancer growth and maintained mouse viability. Although the mice in all groups were similar in size within standard deviation when sacrificed, the mice treated with both inhibitors were slightly larger in general than the untreated controls, 21.3+/−2.9g vs. 20.1+/−4.6g, while those treated with the Mirk inhibitor or RAD001 alone averaged 20.6+/−3.1g and 20.8+/−2.8g, respectively.

As compiled in the Cancer Genome Atlas, the PI3K/Akt/mTOR/p70S6K signaling pathway is frequently deregulated in solid tumors, including pancreatic cancers. Ras proteins directly bind and activate PI3 kinases. Mutant K-ras-driven lung adenocarcinomas undergo stasis and partial regression if the binding between K-ras and the PI3K subunit p110alpha is blocked [38], showing that the PI3K signaling pathway is essential for tumor maintenance initiated by mutant K-ras, at least in a lung adenocarcinoma model. The mTOR inhibitor RAD001 (everolimus) has been approved by the FDA for renal cell carcinoma, giant cell astrocytoma in tuberous sclerosis patients, hormone receptor positive/HER2 negative breast cancer and in neuroendocrine pancreatic cancer [39]. This is a relatively limited range of cancers possibly because mTOR inhibitors upregulate several survival pathways including growth factor receptors. One of the survival proteins upregulated by mTOR inhibition is the Mirk/dyrk1B kinase, through CREB binding sites in the Mirk promoter [27]. RAD001 upregulates Mirk expression, leading to Mirk protein levels elevated up to 10-fold in various pancreatic cancer and ovarian cancer cell lines [27]. We were surprised that RAD001 had such strong effects in the genetic mouse model, as it does not have such effects in patients as a single agent, but the genetic model has not always been predictive.

## CONCLUSIONS

Recent studies have shown that sonic-hedgehog-dependent pancreatic cancer stroma suppresses or restrains pancreatic cancer development, and that Shh-deficient tumors had reduced stromal content, but were more aggressive [36], [37]. Thus the Mirk inhibitor effects of increasing fibroblast growth and subsequent stromal collagen content in the genetic model (Fig.6) may explain why these tumors were not as lethal to their hosts as the untreated tumors (Fig.5A). Thus, the combination of RAD001 with a Mirk kinase inhibitor may warrant further exploration as a possible therapeutic modality.

## MATERIALS & METHODS

Cell lines, media, western blotting, MTT assays, ROS measurements, and transfections were as described [6]. Western blotting was quantitated from autoradiographs using the UN-SCAN-IT gel 5.3 program. All cell lines were purchased from the ATCC in 2009, and their identities confirmed by STR profiles in June, 2014 by Promega through the ATCC. Seventeen short tandem repeat (STR) loci plus the gender determining locus, Amelogenin, were amplified using the commercially available PowerPlex® 18D Kit from Promega. Each cell line sample was processed using the ABI Prism® 3500xl Genetic Analyzer. Data were analyzed using GeneMapper® ID-X v1.2 software (Applied Biosystems). Appropriate positive and negative controls were run and confirmed for each sample submitted. The submitted profiles were an exact match for the Panc1 ATCC human cell line in the ATCC STR database (14 of 14 loci) and the BxPC3 human cell line (12 of 12 loci). In May 2012, short tandem repeat profiling of 15 loci was used to authenticate the TOV21G cell line.

### Mirk/dyrk1B inhibitors

EHT5372 was the gift of Diaxonhit (Paris, France). EHT5372 at 1μM was a stable compound in a human liver microsome study, with 77.4% of the drug remaining after 60 min, with a half-life of over 120 min as assayed by LC-MS [20]. EHT5372 had an IC50 of 0.28nM on the synthetic peptide substrate Dyrktide, and was highly selective in a screen of 339 kinases, including members of the dyrk family [20]. The Chk1 inhibitor LY2603618 and the mTOR inhibitor RAD001 were purchased from Selleck Chemicals. All other reagents were from Sigma.

### Spheroid culture preparation

1 million cells are plated in a 100mm tissue culture dish in 10ml spheroid media and allowed to attach overnight. Cells are then trypsinized, washed twice, then suspended in 10 ml spheroid media (DMEM supplemented with 0.5% FBS) and placed in an ultra-low attachment dish (Fisher), and in 2-3 days spheroid formation occurred. Viable cell numbers in spheroids were determined by metabolism of MTT. All biochemical experiments were repeated at least twice.

### Mouse studies

J:NU athymic mice (Jackson Labs) were injected subcutaneously under the backskin with either 1 million (experiment 1, 4 week old mice) or 10 million viable Panc1 cells (experiment 2, 7 week old mice). When 1 million cells were injected, after 3 weeks palpable tumors were detected, and mice then were subjected to twice weekly 0.1ml intraperitoneal injection with Mirk/dyrk1B inhibitor EHT5372 to give a final concentration of 2, 5 or 10μM, or diluent over a 2-week period. When 10 million cells were injected, after 12 days palpable tumors were detected, and mice were subjected to twice weekly 0.1ml intraperitoneal injection with either Mirk/dyrk1B inhibitor EHT5372 to give a final concentration of 10μM (4mg/kg), gemcitabine to give a final concentration of 25mg/kg, the Chk1 inhibitor LY2603618 to give a final concentration of 4.4 mg/kg, or combinations as indicated, or diluent over a 2 week period. Tumors were excised, weighed and fixed for routine histology and stained for Ki67 expression.

Pdx-1-cre LSL/KrasG12D/Ink4a/Arf null B6 mice were bred from founder mice Pdx-1-cre (B6.FVB- Tg(Ipf1-cre)1Tuv, LSL Kras G12D (B6.129-Kras tm4Tj), Ink4a/Arf null (B6) (B6.129-Cdkn2a tm1Rdp) from the NCI, genotyped at weaning by PCR of tail snips, and then, until 8 weeks of age or death or sacrifice due to ill health, subjected to twice weekly 0.1ml intraperitoneal injection with either 4mg/kg Mirk/dyrk1B inhibitor EHT5372 to give a final concentration of 10μM, 5mg/kg RAD001 to give a final concentration of 5.2μM, the combination, or left untreated. Mice were sacrificed by carbon dioxide inhalation and weighed. Their pancreases were excised and fixed for routine histology andstaining by hematoxylin and eosin, and Masson's trichrome stain. The institutional committee for the humane use of animals approved each protocol.

## SUPPLEMENTARY FIGURE


