# Supplementary material for: Mirk kinase inhibition blocks the in vivo growth of pancreatic cancer cells
Source: Genes Cancer. 2014 Sep;5(9-10):337–47. doi: 10.18632/genesandcancer.29 (PMC4209603; doi:10.18632/genesandcancer.29)
Supplement: Supplementary file 1 [file ganc-05-337-s001.pdf]

Mirk Kinase inhibition blocks the *in vivo* growth of pancreatic cancer cells

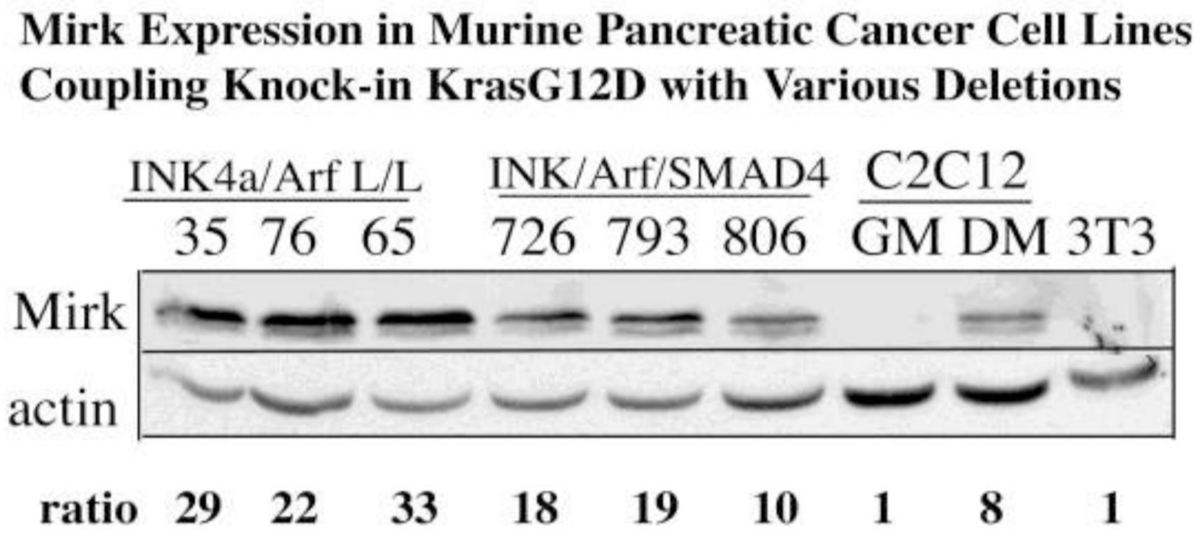

Supplementary Fig. 1: Tumor cell line samples derived from the murine pancreatic cancers were gifts of Dr. N. Bardeesy. They were analyzed for Mirk expression by western blotting. Mirk was expressed in each of three lines (35,76,65) derived from pancreatic cancers in Pdx1-Cre/LSL- KrasG12D/Ink4a/Arf-lox/lox mice, and in each of three lines (726,793,806) from tumors removed from Pdx1-Cre/LSL-KrasG12D/Ink4a/Arf/Smad4-lox/lox mice. The Smad4 gene is deleted in a subset of human pancreatic cancers. The positive control is C2C12 myoblasts which express high levels of Mirk protein when cells are growth in differentiation medium (DM, DMEM plus 2% horse serum) and low levels when grown in growth medium (GM, DMEM plus 7% FBS). The negative control is NIH3T3 cells.
